# Role of Artificial Intelligence in Musculoskeletal Interventions

**DOI:** 10.3390/cancers17101615

**Published:** 2025-05-10

**Authors:** Anuja Dubey, Hasaam Uldin, Zeeshan Khan, Hiten Panchal, Karthikeyan P. Iyengar, Rajesh Botchu

**Affiliations:** 1 Department of Radiology, Healthcare Imaging Centre, Meerut 250001, India; 2 Department of Musculoskeletal Radiology, Royal Orthopedic Hospital, Birmingham B31 2AS, UK; hasaam.uldin1@nhs.net; 3 Department of Orthopedics, Rehman Medical Institute, Peshawar 25000, Pakistan; zeek1978@yahoo.co.uk; 4 Department of Radiology, Sanyapixel Diagnostics, Ahmedabad 380006, India; 5 Department of Orthopedics, Southport and Ormskirk Hospital, Southport L39 2AZ, UK; kartikp31@hotmail.com; 6 Honorary Senior Lecturer, Trauma and Orthopedics MCh Programme, Edge Hill University, Ormskirk L39 4QP, UK; 7 Department of Radiology, NRI Institute of Medical Sciences, Visakhapatnam 531163, India

**Keywords:** artificial intelligence, musculoskeletal imaging, musculoskeletal interventions, machine learning, deep learning, ultrasound-guided interventions, CT-guided interventions, fluoroscopy, radiation dose optimization, precision medicine, robotics, augmented reality

## Abstract

Artificial intelligence (AI) has rapidly emerged as a transformative force in musculoskeletal imaging and interventional radiology. This article explores how AI-based methods—including machine learning (ML) and deep learning (DL)—streamline diagnostic processes, guide interventions, and improve patient outcomes. Key applications discussed include ultrasound-guided procedures for joints, nerves, and tumor-targeted interventions, along with CT-guided biopsies and ablations, and fluoroscopy-guided facet joint and nerve block injections. AI-powered segmentation algorithms, real-time feedback systems, and dose-optimization protocols collectively enable greater precision, operator consistency, and patient safety. In rehabilitation, AI-driven wearables and predictive models facilitate personalized exercise programs that can accelerate recovery and enhance long-term function. While challenges persist—such as data standardization, regulatory hurdles, and clinical adoption—ongoing interdisciplinary collaboration, federated learning models, and the integration of genomic and environmental data hold promise for expanding AI’s capabilities. As personalized medicine continues to advance, AI is poised to refine risk stratification, reduce radiation exposure, and support minimally invasive, patient-specific interventions, ultimately reshaping musculoskeletal care from early detection and diagnosis to individualized treatment and rehabilitation.

## Simple Summary

Artificial Intelligence (AI) is a fundamental aspect of an evolving paradigm shift in radiology. This article outlines how AI-based methods are driving changes in diagnostic and interventional musculoskeletal radiology with a wide range of specific applications discussed including procedures involving ultrasound, CT, and fluoroscopy. Methods of utilizing AI to optimize the patient and practitioner experience such as feedback systems, dose-optimization, and segmentation algorithms are reviewed. These changes will play a significant role in shaping the rapidly changing landscape of musculoskeletal radiology.

## 1. Introduction

Artificial intelligence (AI) refers to a range of computational methods capable of performing tasks that replicate human cognition, including learning, reasoning, and decision-making [1,2]. These methods span from simple, rule-based algorithms to more advanced machine learning (ML) and deep learning (DL) models. Through continuous analysis of new data, AI systems can refine their performance without requiring explicit reprogramming [1,2].

AI encompasses various specialized branches. Natural language processing (NLP) enables computers to interpret and generate human language; computer vision focuses on analyzing and interpreting visual information; and robotics supports precision-driven tasks in physical environments [3,4]. Owing to this versatility, AI adoption has expanded across multiple industries, especially healthcare, where its impact is seen in diagnostic imaging and interventional radiology. Recently, musculoskeletal imaging and associated procedures have undergone a notable shift due to AI-driven enhancements, delivering improved diagnostic accuracy, targeted therapies, and better health outcomes [5].

In musculoskeletal imaging and interventions, AI is reshaping traditional workflows by refining imaging acquisition, interpretation, and clinical integration. Advanced deep learning models expedite MRI and CT scanning while maintaining high diagnostic quality. Segmentation algorithms automatically identify essential structures—bones, cartilage, and tumors—offering critical guidance to clinicians. Furthermore, AI-based radiomic analysis supports early diagnosis of osteoporosis, osteoarthritis, and musculoskeletal neoplasms, potentially surpassing established diagnostic methods [6].

Beyond image processing, AI enhances radiology operations by generating automated reports, highlighting urgent or high-risk findings, and reducing radiologists’ workload. It has also improved the accuracy of fracture detection in plain radiographs, minimizing misinterpretation and expediting treatment. Integrating AI in musculoskeletal imaging and procedures paves the way for precision-oriented medical care, streamlining operations and improving patient experiences. Facilities implementing AI strategies often see fewer workflow delays, resulting in overall greater efficiency [7,8,9,10,11,12]. ChatGPT 4o is an advanced, multimodal AI tool integrating text, vision, and audio. It demonstrates high accuracy in communicating the risks and benefits of musculoskeletal interventions and orthopedic procedures. Further prospective studies are necessary to validate its effectiveness in medical consent [13].

## 2. The Role of AI in Musculoskeletal Interventions

Minimally invasive interventions in the musculoskeletal system have transformed the management of orthopedic and rheumatologic disorders, typically leading to shorter recovery periods and lower complication rates [5,14,15]. Nevertheless, these procedures can still be hindered by operator variability, suboptimal imaging, and the logistical hurdles of real-time navigation—issues that can diminish success rates and prolong procedure times [16].

AI helps address these challenges by improving imaging clarity, automating segmentation, and enhancing procedural accuracy [2,3]. Deep learning algorithms, in particular, show great promise in guiding needles precisely, planning ablation zones, and predicting procedural outcomes. Breakthroughs in convolutional neural networks (CNNs) and recurrent neural networks (RNNs) enable more refined interpretations of both image-based and temporal data, thus further improving musculoskeletal interventions. The following sections detail the evolving influence of AI in these procedures and explore current benefits and future developments.

## 3. AI Applications in Musculoskeletal Interventions

### 3.1. AI in Ultrasound-Guided Procedures

#### 3.1.1. Joint and Bursal Injections

Ultrasound-guided injections into joints and bursae help control pain and inflammation in conditions like osteoarthritis and bursitis. AI adds value by automatically marking relevant anatomical features, adjusting needle trajectories, and optimizing probe placement. Deep learning-based segmentation techniques make it easier for clinicians to distinguish the joint space from nearby soft tissues, ensuring accurate delivery of medications (Table 1) [17].

#### 3.1.2. PRP Injections for Cartilage Injuries

Platelet-rich plasma (PRP) injections are increasingly used to treat cartilage lesions and chronic joint issues. AI-assisted imaging helps target cartilage defects precisely and select optimal injection sites. Moreover, predictive models factor in patient demographics, clinical markers, and lifestyle variables to estimate therapeutic success, enabling customized strategies aimed at quicker symptom resolution and better cartilage repair [17].

#### 3.1.3. Ultrasound-Guided Tumor Biopsy

AI-enhanced ultrasound significantly reduces operator dependence in tumor biopsies. ML techniques can categorize echogenic properties, aiding physicians in differentiating malignant from benign lesions [18,19,20]. By automating trajectory planning, AI lowers the risk of insufficient sampling. This becomes increasingly relevant as understanding of tumor heterogeneity grows; AI pinpoints the most suspicious lesion regions, thus increasing diagnostic accuracy and easing patient anxiety.

#### 3.1.4. Nerve Hydro-Dissection and Hydrodilatation for Adhesive Capsulitis

AI refines ultrasound-guided nerve hydrodissection by identifying nerve compression patterns and optimizing the agent’s distribution [21]. It also enhances hydrodilatation procedures for adhesive capsulitis by automating capsule segmentation, refining injectate volume, and providing real-time visualization of capsule expansion. Together, these advances facilitate better pain management, improved range of motion, and more accurate rehabilitation scheduling.

#### 3.1.5. AI in Dry Needling for Tendinosis

Using AI for imaging guidance in chronic tendinosis helps pinpoint affected tendon areas while avoiding nearby neurovascular structures. Deep learning techniques can analyze elastography data and identify fibrotic tissue in need of treatment, promoting precise and effective needling [17]. Research indicates that combining AI-driven dry needling with adjunctive therapies, such as PRP or shockwave therapy, might accelerate healing and boost tissue renewal [22,23].

#### 3.1.6. AI in Diagnosis and Treatment of Plantar Fasciitis

Plantar fasciitis (PF) is a frequent cause of heel pain stemming from repetitive stress and micro-tearing of the fascia, leading to tissue thickening and chronic inflammation. Precision is key for detecting and addressing PF, and AI shows strong potential in this domain.

AI tools can analyze ultrasound scans to measure fascial thickness, locate microtears, and estimate inflammation severity with greater precision, enabling more accurate PF diagnosis. In interventions, AI improves ultrasound imaging by boosting contrast and reducing noise, thereby helping clinicians target compromised areas more accurately. This can enhance outcomes in methods such as dry needling or percutaneous needle electrolysis [23].

Additionally, AI-based approaches that integrate biologic treatments—like PRP—and customized orthotic support tailor interventions to the individual, which may shorten recovery timelines. Overall, AI-driven diagnosis and therapy mark a significant improvement in PF management, offering precision, efficiency, and personalization.

#### 3.1.7. AI in Ultrasound-Guided Nerve Blocks for Musculoskeletal Interventions

AI profoundly impacts ultrasound-guided nerve blocks by increasing precision, effectiveness, and safety. By harnessing deep learning and computer-aided detection models, AI automatically highlights nerve structures in real time, improving needle placement and reducing the burden on operator expertise [24,25].

This is especially important for brachial plexus blocks, where precise nerve identification is essential. AI-based mapping delineates nerves, vessels, and muscles, lowering risks of vascular puncture or nerve injury. In broader musculoskeletal interventions, ultrasound-guided nerve blocks help manage pain and enable minimally invasive procedures like tendon repair and synovial biopsies. AI-driven needle tracking refines real-time imaging, ensuring anesthetic agents are precisely placed, particularly in complex entrapments or post-operative scenarios.

#### 3.1.8. AI in Ultrasound-Guided Detection of Synovitis and Guiding Synovial Biopsy

AI is revolutionizing how synovitis is identified and characterized, leading to more accurate diagnoses and targeted biopsies. Convolutional neural networks (CNNs) can achieve high accuracy—over 90% in some studies—in grading synovitis on ultrasound [26]. Another review underscores AI’s extensive applications in musculoskeletal ultrasound, highlighting its effectiveness for inflammation assessment.

Beyond simple detection, AI differentiates various types of synovitis by analyzing histological findings, thereby distinguishing osteoarthritis from autoimmune conditions like rheumatoid arthritis. By integrating with imaging devices, AI also helps guide biopsies to areas of maximum inflammation, improving diagnostic yield and reducing patient discomfort.

### 3.2. AI in CT-Guided Musculoskeletal Interventions

#### 3.2.1. CT-Guided Biopsy

AI-based segmentation in CT scans offers clearer lesion visualization, aiding needle placement for biopsies and minimizing complication risks [27]. In addition, deep learning models identify the safest biopsy route while considering vascular or neural structures. Spectral CT imaging integrated with AI also promises more granular information on tissue makeup, paving the way for greater diagnostic precision and optimized therapy.

#### 3.2.2. Radiofrequency Ablation (RFA) for Osteoid Osteoma

Thermal mapping algorithms enhance RFA for osteoid osteoma, ensuring thorough lesion ablation while sparing healthy tissue [28]. Real-time monitoring of heat distribution helps verify complete ablation, lowering recurrence risk. Since osteoid osteomas can appear in challenging anatomical locations, AI-based segmentation and thermal feedback reduce complications and accelerate recovery.

## 4. AI in Fluoroscopy-Guided Interventions

### 4.1. Facet-Joint and Nerve-Block Injections

Artificial intelligence software has numerous applications. In planning, this includes being able to review each real-time frame in fluoroscopy-guided spine interventions, outline pedicles and facet clefts, and overlay a color-coded path indicating the optimal needle path. Since the best route can therefore become apparent, the operator can advance with fewer fluoroscopic acquisitions, reducing total scan time and patient and team radiation [29]. As the needle traverses, motion-correction algorithms can track its tip and adjust the path when respiration or table drift would otherwise compromise accuracy. This can help to maintain needle stability, essential for lengthy procedures where small shifts can jeopardize success. At the same time, intelligent exposure control constricts the beam to the active field and reduces the pulse rate in the periods of rest, gaining up to 40% dose savings without compromising image clarity.

### 4.2. Arthrography

Artificial intelligence currently functions as an advanced co-pilot for fluoroscopy-guided arthrography [30]. Prior to capturing the initial image, AI can highlight osseous contours and capsular recesses on the scout view, determining the safest needle entry point and ideal depth—particularly advantageous when osteophytes or joint-space narrowing conceal traditional landmarks [31]. While advancing the needle, computer-vision software monitors the tip in real time, superimposing a colored trajectory that facilitates fast course correction if it deviates extra-articularly. Adaptive algorithms consistently optimize pulse rate and collimation, reducing radiation exposure while maintaining visual clarity [14]. As contrast is administered, a specialized AI algorithm tracks its dispersion in real-time and generates an alert if opacification does not remain intra-articular, thereby minimizing the need for repeated injections and repositioning. Following the operation, the system autonomously logs contrast volume, image quantity, and any irregular capsular capacity, offering actionable insights for future cases and quality assessments. By transferring essential responsibilities from operator intuition to data-driven insights, AI enhances the speed, safety, and reproducibility of fluoroscopic arthrography.

### 4.3. Percutaneous Biopsy

Artificial intelligence plays an increasingly valuable role in enhancing the precision, safety, and efficiency of fluoroscopy-guided bone biopsies. AI-based planning systems begin by analyzing pre-procedural CT or MRI scans to automatically identify bone lesions and segment critical surrounding anatomy such as nerves, vessels, or bowel loops. Using this anatomical data, AI computes an optimal needle trajectory that avoids sensitive or high-risk anatomical structures while targeting the center of the lesion. Once fluoroscopy is initiated, this planned path is superimposed onto the real-time fluoroscopic image, providing intuitive visual guidance for the radiologist. This overlay allows for more accurate needle placement, reduces the number of corrective passes, and minimizes tissue trauma [14,32].

Real-time computer vision algorithms can track the biopsy needle during advancement, alerting operators if the trajectory deviates from the planned path. Additionally, AI-controlled fluoroscopy systems can automatically adjust image acquisition parameters such as pulse rate and field-of-view, thereby reducing radiation exposure to both patients and staff without compromising visibility [14].

## 5. AI in Musculoskeletal Rehabilitation

**Figure 1:**
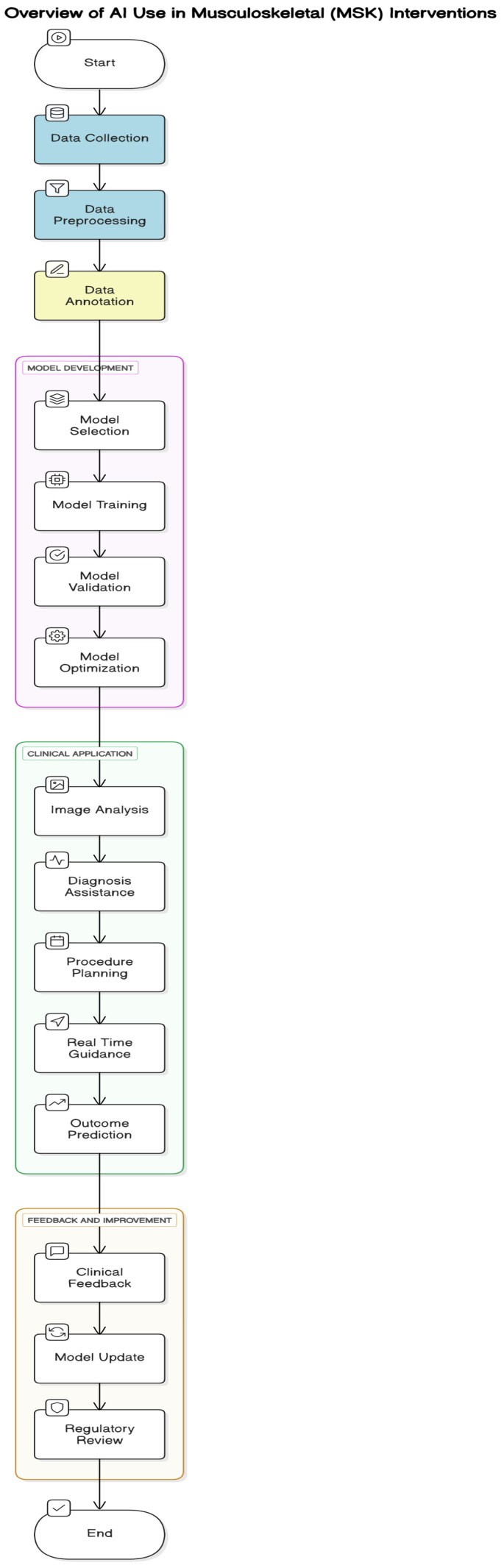
Overview of AI use in Musculoskeletal (MSK) interventions.

Artificial intelligence is transforming musculoskeletal rehabilitation by making recovery more personalized and efficient. After surgeries or injuries, wearable devices and smartphone apps can track how a patient moves, how muscles are used, and how well exercises are performed [33]. AI analyzes these data to check progress, identify movement problems, and suggest customized exercise plans. For instance, the Persona IQ^®^ smart knee implant collects motion data post-surgery, allowing healthcare providers to monitor recovery remotely and adjust treatment plans accordingly. Similarly, the Reflex Health app uses AI-powered pose estimation to guide shoulder rehabilitation exercises, providing real-time feedback and tracking range of motion improvements. By integrating rehabilitation outcomes with imaging data, clinicians can better understand healing progress and tailor interventions more effectively. This AI-driven approach (Figure 1) not only accelerates recovery and improves physical function but also enables remote monitoring, reducing the need for frequent hospital visits.

## 6. Advantages of AI over Conventional Techniques

### 6.1. Improved Accuracy and Precision

Artificial intelligence transforms procedural precision by taking advantage of cutting-edge technologies like automated segmentation, real-time feedback loops, and advanced path-planning algorithms (Table 2). These technologies greatly reduce variability linked to human operator experience, thereby providing consistent results in procedures such as injections, biopsies, and ablations. Automated segmentation uses deep learning to thoroughly examine medical imaging, defining anatomical structures and pathological areas with accuracy, customized to every patient’s individual anatomy. Real-time feedback loops continuously track tool location and tissue motion, allowing instantaneous correction for deviations due to patient movement or tissue deformation, improving safety. Path-planning algorithms compute optimal needle or probe trajectories, steering clear of vital structures such as blood vessels or nerves while optimizing insertion force or ablation energy as a function of tissue properties. By tailoring interventions to consider individual anatomical and tissue differences, AI reduces complications, enhances therapeutic effectiveness, and provides safer, more consistent procedural results [34].

### 6.2. Increased Procedural Efficiency

AI optimizes medical interventions by eliminating time-consuming processes, including image analysis and trajectory planning, thus enhancing procedural efficiency [3,9]. By quickly processing imaging data and producing accurate plans for needle placement or ablation zones, AI decreases the length of procedures, which is especially beneficial in high-volume clinical settings. This productivity means shorter times under anesthesia, reduced discomfort for patients, and a diminished risk of complications, ultimately enhancing patient satisfaction. Furthermore, AI’s ability to preprocess imaging and suggest optimal procedural steps allows clinicians to focus on critical decision-making, reducing cognitive load and enhancing workflow. In settings where time is a critical factor, such as emergency interventions, AI-driven automation ensures swift and accurate execution, contributing to better resource utilization and improved patient experiences.

### 6.3. Radiation Exposure Reduction

Radiation remains a concern for interventions relying on fluoroscopy or CT. AI’s dose-optimization algorithms help maintain sufficient image quality at lower radiation levels by modifying exposure based on patient anatomy and procedure requirements [27,29]. By shortening fluoroscopy time and introducing dose-saving strategies, AI reduces the radiation burden on healthcare workers and patients.

In CT-guided procedures, AI can facilitate low-dose scans, offering advanced image reconstruction and spectral imaging. As a result, imaging time and overall radiation exposure are lowered without sacrificing diagnostic clarity. AI-enhanced ultrasound may also substitute some radiographic assessments, further cutting reliance on ionizing radiation—benefiting patients requiring repeated imaging by protecting them from cumulative exposure. This is especially valuable for patients with chronic illnesses that need regular imaging.

### 6.4. Predictive Analytics for Tailored Treatment Strategies

AI applies predictive analytics to provide tailored treatment plans through the integration of varied patient data, such as medical history, genetic information, and risk factors [18]. Machine learning algorithms process large datasets to predict procedural outcomes, detect possible complications, and suggest optimized intervention plans. As the algorithms improve with ongoing learning, they offer increasingly precise real-time information, allowing clinicians to dynamically adjust care plans.

For instance, AI can predict the probability of adverse events under a biopsy or ablation so pre-emptive corrections can be made to improve safety. By applying individualized intervention based on patient profiles, predictive analytics enhance procedure success rates, reduce risks, and facilitate precision medicine. Empowering clinicians through data-driven methods, predictive analytics enables them to make informed choices and ensure that treatments are not only effective but also tailored to the specific needs of each patient.

### 6.5. Advanced Fusion Imaging

Artificial intelligence-powered fusion imaging combines information from multiple imaging modalities, such as magnetic resonance imaging (MRI), computed tomography (CT), and ultrasound, to create comprehensive, multi-dimensional representations of anatomical structures. This integrated approach is particularly valuable in the diagnosis and treatment of complex musculoskeletal conditions and interventions—such as spinal procedures or large joint injections—where precise anatomical visualization is critical [1,2,3,4].

By offering a holistic and detailed view of the target region, AI-enhanced fusion imaging improves diagnostic accuracy and procedural planning. It allows clinicians to better assess tissue characteristics, align needle trajectories, and identify critical structures to avoid, thereby enhancing the safety and efficacy of image-guided interventions. This advanced visualization capability represents a major advancement in musculoskeletal care, supporting more precise, confident, and minimally invasive procedures.

### 6.6. Integration with Robotics and Augmented Reality (AR)

AI improves procedural accuracy by integrating with robotic systems and augmented reality (AR) solutions, eliminating key steps and minimizing human error [5,14]. In robotic-assisted procedures, AI can direct needle guidance, ablation placement, or implantation with sub-millimeter precision, especially in sensitive areas such as the spine or brain. Dynamic visual cues via real-time AR overlays give surgeons instant feedback, superimposing vital anatomical information onto the surgical site, which enhances confidence and precision. This combination of AI, robotics, and AR reduces operator fatigue, simplifies intricate procedures, and provides uniform results. As these technologies become more advanced, clinicians will increasingly use AI-driven robotic systems to perform complex tasks under human oversight, leading the way for semi-autonomous interventions that merge technological accuracy with clinical acumen.

### 6.7. Virtual Biopsy and Radiomic Profiling for Non-Invasive Diagnostics

Artificial intelligence-driven radiomic analysis and virtual biopsy methods provide non-invasive solutions to conventional tissue characterization by deriving complex patterns from imaging data, including pixel intensity, texture, and shape [14]. These approaches allow AI to discern subtle pathological alterations, potentially serving as surrogates for risky invasive biopsies. By integrating radiomic information with molecular and genomic information, AI allows clinicians to perform exhaustively detailed disease profiling, making it possible to stratify risks and formulate treatment plans with precision. For example, AI is able to identify early microstructural alterations in tissues, helping in pre-emptive diagnosis of cancer or degenerative diseases. As these systems evolve, they could optimize early intervention tactics, decrease the demand for invasive interventions, and enhance patient outcomes by facilitating timely, tailored therapeutic choices based on non-invasive diagnostic information.

## 7. Practical Clinical Considerations

### 7.1. Multidisciplinary Teamwork

The effective deployment of AI in clinical environments relies on strong collaboration between various professionals, such as data scientists, radiologists, orthopedic surgeons, and IT experts. This multidisciplinary collaboration ensures that AI tools are developed to solve salient clinical problems using complete and representative datasets [1,2,5]. Through open communication and collaborative expertise, teams can create AI solutions that are technically valid and clinically applicable, ultimately improving diagnostic accuracy and treatment outcomes.

### 7.2. Real-Time Adaptation and Closed-Loop Mechanisms

The emergence of closed-loop systems is a development in AI-guided medical procedures. Closed-loop systems allow immediate adjustment of procedural parameters based on real-time imaging feedback [14,15]. In an ablation procedure, for instance, if the AI realizes there is not enough coverage, it will suggest accurate repositioning or energy optimization [28]. This dynamic adaptability improves procedural accuracy, reduces risks, and opens up the possibility for more automated and streamlined clinical processes.

### 7.3. Cost Implications

The deployment of AI in healthcare comes with high initial costs, such as software licenses, training programs for personnel, and infrastructure investment. Yet these expenditures can provide substantial long-term gains, including enhanced patient throughput, fewer medical errors, and optimized care delivery [10,11]. Through the enhancement of operational efficiency and patient outcomes, AI would eventually pay back initial investments and provide cost-saving solutions that make healthcare systems more sustainable.

### 7.4. Ethical and Legal Considerations

With AI playing a greater role in clinical decision-making, clear ethical and legal standards are the top priority. Questions of liability in the event of errors due to AI must be settled in order to hold entities responsible while safeguarding patients and healthcare workers. Regulatory authorities must create strong frameworks to control the application of increasingly autonomous AI systems, reconciling innovation with patient protection and trust. These standards will be essential to ensuring ethical practices and building trust in AI-based healthcare [13,14].

## 8. Challenges, Limitations and Future Directions in AI for Musculoskeletal Interventions

### 8.1. Challenges of AI Implementation in Musculoskeletal Care

Despite its notable advantages, AI in musculoskeletal care faces some hurdles. Data standardization remains a significant issue, as imaging protocols and equipment vary across centers [9,11]. Regulatory approvals can also slow clinical deployment, as continuous-learning algorithms require careful oversight to ensure safety [13,14].

Clinician acceptance is another hurdle. Simple, intuitive AI interfaces and proper training programs are essential for successful integration [10,11]. Handling diverse patient profiles—such as those with implants or rare disorders—requires continual AI refinement based on real-world datasets [9,11,16]. Additionally, patient-data security is paramount, and federated learning strategies can help maintain confidentiality.

### 8.2. Limitation of Current AI Tool for Risk of Overreliance

One of the main concerns with using AI in musculoskeletal interventions is the risk of over-reliance. While these tools can be incredibly helpful—like analyzing images, planning needle paths, or guiding procedures—they are not perfect. Sometimes, AI might make mistakes if the data it was trained on do not match real-life situations or if a patient’s anatomy is unusual. There is also a chance it might miss important details that an experienced clinician would catch. If clinicians depend too heavily on AI without double-checking or using their own judgment, it could lead to errors in diagnosis or treatment. AI also does not always adapt well to unexpected changes during a procedure, like a patient shifting or tissues moving. Therefore, while AI can be a great assistant, it is important to remember that it is a tool and the final decisions should always come from the trained hands and eyes of the medical team [35].

### 8.3. Future Trends in AI

AI is moving toward integrative analytics that combine patient history and imaging, often employing natural language processing for advanced diagnostic applications [1,2,3,4,6]. Wearables may generate real-time data on joint function and muscle activity, enabling personalized therapy. Telemedicine is also expanding, allowing experts to supervise procedures remotely, thus improving access in underserved regions [5,6].

Progress in AI for musculoskeletal interventions hinges on strong collaboration among healthcare facilities, researchers, and technology firms [1,5,11]. By developing secure, standardized data-sharing channels, AI tools will become more robust and inclusive. As personalized medicine evolves, AI will increasingly integrate genomic and environmental data to tailor interventions. Federated learning and explainable AI may further address concerns about data security and regulatory compliance.

## 9. Future Prospects of AI-Driven Musculoskeletal Care

### 9.1. Biological Augmentation

When combined with next-generation regenerative therapies—like stem cells or gene-editing techniques—AI can help deliver these treatments precisely and adjust dosages in real time. Imaging feedback might prompt additional injections if healing lags, supporting personalized care strategies [15,21].

### 9.2. Tele-Robotic Interventions

AI and robotics can bridge gaps in rural or underserved communities by allowing specialists to conduct procedures remotely. This tele-robotic approach significantly broadens access to high-level care and reduces healthcare disparities [5,27,29].

### 9.3. Integration with Genomic Data

AI platforms that integrate imaging findings with genomics can predict a patient’s response to treatments ranging from conservative measures to surgical options like joint replacement [18]. Customizing therapy could shorten recovery times and raise success rates.

## 10. Conclusions

AI is transforming musculoskeletal interventional radiology by offering more precise procedures, increased workflow efficiency, and stronger clinical outcomes. Ultrasound, CT, and fluoroscopy all stand to benefit, as AI refines accuracy, moderates radiation exposure, and yields reproducible results. Moving forward, AI will likely intertwine with robotics, augmented reality, and predictive modeling, continuing to drive the advancement of precision musculoskeletal care. This evolution promises more targeted, data-informed treatments for complex musculoskeletal cases.

Although certain challenges—data exchange, regulatory clearance, and clinical integration—remain, ongoing research and interdisciplinary collaboration are working to resolve these barriers. As AI gains traction, musculoskeletal treatments will likely become even more personalized, efficient, and effective. Combined with biologic therapies, genetics research, and remote care, AI has the potential to reshape every facet of musculoskeletal practice, from early diagnosis to final rehabilitation.

Ultimately, AI’s development points to safer interventions, faster recovery, and fully individualized therapies. The resulting progress will greatly enhance patient experiences in both orthopedic and rheumatology care. By capitalizing on AI’s capabilities, the musculoskeletal field can evolve into a more patient-centric and technology-driven discipline, influencing the next era in medical management.

## Figures and Tables

**Table 1:** Applications and benefits of AI in specific musculoskeletal interventions.

Intervention Type	Additional Value of AI	Key Benefits
**Ultrasound-guided joint injections**	Automated anatomical structure identification Improved needle visualization	Increased accuracy and efficacy Reduced procedural time
**PRP injections for cartilage repair**	Predictive modeling for therapeutic outcomes	Increased accuracy and efficacy Reduced procedural time Personalized treatment
**Nerve blocks and hydro-dissection**	AI-driven nerve segmentation and mapping Optimized injectate distribution	Reduced operator dependency Improved accuracy and optimization of site, yielding improved pain relief Reduced procedural variability
**Dry needling (for tendinosis)**	Improved tissue differentiation with the use of elastography AI-assisted targeting	Enhanced precision, better healing response
**Ultrasound-guided biopsy**	Lesion differentiation Improved needle guidance and path optimization	Improved needle accuracy and minimization of sampling errors Reduced procedural time Safer access to the lesion
**CT-guided Biopsy**	Lesion differentiation Needle guidance and path optimization	Improved accuracy and tissue sampling Lower complication rates Reduced procedural time
**Radiofrequency ablation (ultrasound/CT)**	Thermal mapping algorithms AI-guided ablation monitoring	Reduced recurrence Targeted tissue destruction with reduction of collateral damage
**Fluoroscopy-guided nerve blocks**	Automated anatomical landmark detection Anatomical correlation with other available imaging Motion-correction algorithms	Improved efficiency Minimized radiation exposure

**Table 2:** General advantages of AI over conventional techniques in musculoskeletal radiology.

Aspect	Traditional Approach	AI-Enhanced Approach	Impact of AI Use on Clinical Practice
**Accuracy and precision**	Operator-dependent Subjective interpretation	Automated segmentation Real-time feedback	Reduced variability Improved safety and accuracy
**Interventional procedural efficiency**	Manual planning Longer intervention times Variable needle trajectories	AI-assisted automation Optimized workflow	Shorter procedures Reduced errors
**Radiation exposure**	Operator-dependent fluoroscopy/CT usage	Dose optimization algorithms Reduced procedural time	Lower radiation burden for patients and staff
**Predictive analytics**	Experience-based predictions	Data-driven, personalized risk assessments	Improved patient-specific planning
**Fusion imaging**	Limited to single-modality views	AI-driven multimodal integration	Enhanced visualization for complex cases by combining imaging modalities
**Integration with robotics**	Manual execution with robot Potential human errors	AI-assisted robotic precision	Reduced operator fatigue Increased safety

## References

[1] Tang X. (2019). The role of artificial intelligence in medical imaging research. BJR Open.

[2] Chartrand G., Cheng P.M., Vorontsov E., Drozdzal M., Turcotte S., Pal C.J., Kadoury S., Tang A. (2017). Deep learning: A primer for radiologists. RadioGraphics.

[3] Lee J.-G., Jun S., Cho Y.-W., Lee H., Kim G.B., Seo J.B., Kim N. (2017). Deep learning in medical imaging: General overview. Korean J. Radiol..

[4] Erickson B.J., Korfiatis P., Akkus Z., Kline T.L. (2017). Machine learning for medical imaging. Radiographics.

[5] Guermazi A., Omoumi P., Tordjman M., Fritz J., Kijowski R., Regnard N.-E., Carrino J., Kahn C.E., Knoll F., Rueckert D. (2024). How AI May Transform Musculoskeletal Imaging. Radiology.

[6] Shin Y., Kim S., Lee Y.H. (2021). AI musculoskeletal clinical applications: How can AI increase my day-to-day efficiency?. Skelet. Radiol..

[7] Bhandari A. (2024). Revolutionizing Radiology With Artificial Intelligence. Cureus.

[8] Chen M., Wang Y., Wang Q., Shi J., Wang H., Ye Z., Xue P., Qiao Y. (2024). Impact of human and artificial intelligence collaboration on workload reduction in medical image interpretation. npj Digit. Med..

[9] Tong M.W., Zhou J., Akkaya Z., Majumdar S., Bhattacharjee R. (2024). Artificial intelligence in musculoskeletal applications: A primer for radiologists. Diagn. Interv. Radiol..

[10] Yi P.H., Garner H.W., Hirschmann A., Jacobson J.A., Omoumi P., Oh K., Zech J.R., Lee Y.H. (2024). Clinical Applications, Challenges, and Recommendations for Artificial Intelligence in Musculoskeletal and Soft-Tissue Ultrasound: AJR Expert Panel Narrative Review. Am. J. Roentgenol..

[11] Gitto S., Serpi F., Albano D., Risoleo G., Fusco S., Messina C., Sconfienza L.M. (2024). AI applications in musculoskeletal imaging: A narrative review. Eur. Radiol. Exp..

[12] Shin Y., Yang J., Lee Y.H., Kim S. (2021). Artificial intelligence in musculoskeletal ultrasound imaging. Ultrasonography.

[13] Hussein A., Hussein M., Shirodkar K., Kanani A., Iyengar K.P., Botchu R. (2025). How does ChatGPT 4omni perform in consenting for common orthopedic and musculoskeletal interventional procedures?. J. Arthrosc. Surg. Sports Med..

[14] Glielmo P., Fusco S., Gitto S., Zantonelli G., Albano D., Messina C., Sconfienza L.M., Mauri G. (2024). Artificial intelligence in interventional radiology: State of the art. Eur. Radiol. Exp..

[15] Hattori S., Saggar R., Heidinger E., Qi A., Mullen J., Fee B., Brown C.L., Canton S.P., Scott D., Hogan M.V. (2024). Advances in Ultrasound-Guided Surgery and Artificial Intelligence Applications in Musculoskeletal Diseases. Diagnostics.

[16] Ajmera P., Kharat A., Botchu R., Gupta H., Kulkarni V. (2021). Real-world analysis of artificial intelligence in musculoskeletal trauma. J. Clin. Orthop. Trauma.

[17] Dinescu S.C., Stoica D., Bita C.E., Nicoara A.-I., Cirstei M., Staiculesc M.-A., Vreju F. (2023). Applications of artificial intelligence in musculoskeletal ultrasound: Narrative review. Front. Med..

[18] Isaac A., E Klontzas M., Dalili D., Akdogan A.I., Fawzi M., Gugliemi G., Filippiadis D. (2025). Revolutionising Osseous Biopsy: The Impact of Artificial Intelligence in the Era of Personalized Medicine. Br. J. Radiol..

[19] Brunetti N., Calabrese M., Martinoli C., Tagliafico A.S. (2022). Artificial Intelligence in Breast Ultrasound: From Diagnosis to Prognosis—A Rapid Review. Diagnostics.

[20] Lacroix M., Aouad T., Feydy J., Biau D., Larousserie F., Fournier L., Feydy A. (2022). Artificial intelligence in musculoskeletal oncology imaging: A critical review of current applications. Diagn. Interv. Imaging.

[21] Sveva V., Farì G., Fai A., Savina A., Viva M.G., Agostini F., Ranieri M., Megna M., Mangone M., Paoloni M. (2024). Safety and Efficacy of Ultrasound-Guided Perineural Hydrodissection as a Minimally Invasive Treatment in Carpal Tunnel Syndrome: A Systematic Review. J. Pers. Med..

[22] Butts R., Dunning J., Serafino C. (2021). Dry needling strategies for musculoskeletal conditions: Do the number of needles and needle retention time matter? A narrative literature review. J. Bodyw. Mov. Ther..

[23] Yin M., Ma J., Xu J., Li L., Chen G., Sun Z., Liu Y., He S., Ye J., Mo W. (2019). Use of artificial neural networks to identify the predictive factors of extracorporeal shock wave therapy treating patients with chronic plantar fasciitis. Sci. Rep..

[24] Bowness J., Varsou O., Turbitt L., Laurent D.B. (2021). Identifying anatomical structures on ultrasound: Assistive artificial intelligence in ultrasound-guided regional anesthesia. Clin. Anat..

[25] Finnoff J.T., Hall M.M., Adams E., Berkoff D., Concoff A.L., Dexter W., Smith J. (2015). American Medical Society for Sports Medicine (AMSSM) Position Statement: Interventional Musculoskeletal Ultrasound in Sports Medicine. PMR.

[26] Stoel B. (2020). Use of artificial intelligence in imaging in rheumatology—Current status and future perspectives. RMD Open.

[27] Sato T., Kawai T., Shimohira M., Ohta K., Suzuki K., Nakayama K., Takikawa J., Kawaguchi T., Urano M., Ng K.W. (2025). Robot-Assisted CT-Guided Biopsy with an Artificial Intelligence–Based Needle-Path Generator: An Experimental Evaluation Using a Phantom Model. J. Vasc. Interv. Radiol..

[28] De Filippo M., Russo U., Papapietro V.R., Ceccarelli F., Pogliacomi F., Vaienti E., Piccolo C., Capasso R., Sica A., Cioce F. (2018). Radiofrequency ablation of osteoid osteoma. Acta Biomed..

[29] Gao C., Phalen H., Margalit A., Ma J.H., Ku P.-C., Unberath M., Taylor R.H., Jain A., Armand M. (2022). Fluoroscopy-Guided Robotic System for Transforaminal Lumbar Epidural Injections. IEEE Trans. Med. Robot. Bionics.

[30] Malhotra G., Hansford B.G., Felcher C., Wuerfel K.A., Yablon C.M. (2022). Fluoroscopic-guided procedures of the lower extremity. Skelet. Radiol..

[31] Croci E., Hess H., Warmuth F., Künzler M., Börlin S., Baumgartner D., Müller A.M., Gerber K., Mündermann A. (2023). Fully automatic algorithm for detecting and tracking anatomical shoulder landmarks on fluoroscopy images with artificial intelligence. Eur. Radiol..

[32] Braak S.J., van Strijen M.J.L., van Leersum M., van Es H.W., van Heesewijk J.P.M. (2010). Real-Time 3D Fluoroscopy Guidance During Needle Interventions: Technique, Accuracy, and Feasibility. Am. J. Roentgenol..

[33] Soer R. (2023). Artificial intelligence in musculoskeletal rehabilitation. J. Back Musculoskelet. Rehabil..

[34] Román-Belmonte J.M., De la Corte-Rodríguez H., Rodríguez-Merchán E.C. (2021). Artificial intelligence in musculoskeletal conditions. Front. Biosci. (Landmark Ed.).

[35] Bicer E.K., Fangerau H., Sur H. (2023). Artifical intelligence use in orthopedics: An ethical point of view. EFORT Open Rev..

